# Deficiency of myeloid-related proteins 8 and 14 (Mrp8/Mrp14) does not block inflammaging but prevents steatosis

**DOI:** 10.18632/oncotarget.9550

**Published:** 2016-05-21

**Authors:** William R. Swindell, Xianying Xing, Yi Fritz, Doina Diaconu, Daniel I. Simon, Nicole L. Ward, Johann E. Gudjonsson

**Affiliations:** ^1^ Ohio University Heritage College of Osteopathic Medicine, Athens, OH, USA; ^2^ Department of Dermatology, University of Michigan, Ann Arbor, MI, USA; ^3^ Department of Dermatology, Case Western Reserve University, Cleveland, OH, USA; ^4^ Harrington Heart and Vascular Institute, University Hospitals Case Medical Center, Case Western Reserve University School of Medicine, Cleveland, OH, USA; ^5^ The Murdough Family Center for Psoriasis, Case Western Reserve University, Cleveland, OH, USA

**Keywords:** B-cell, calgranulin, calprotectin, microarray, S100a8, Gerotarget

## Abstract

The Mrp8 and Mrp14 proteins (calprotectin) accumulate within tissues during aging and may contribute to chronic inflammation. To address this possibility, we evaluated female calprotectin-deficient Mrp14-KO and wild-type (WT) mice at 5 and 24 months of age. However, there was no evidence that age-related inflammation is blunted in KO mice. Inflammation markers were in fact elevated in livers from old KO mice, and microarray analysis revealed more consistent elevation of genes specifically expressed by B-cells and T-cells. Adipose-specific genes, however, were less consistently elevated in aged KO mice, suggesting an anti-steatosis effect of Mrp8/14 deficiency. Consistent with this, genes decreased by the anti-steatosis agent SRT1720 were decreased in old KO compared to old WT mice. Expression of lipid metabolism genes was altered in KO mice at 5 months of age, along with genes associated with development, biosynthesis and immunity. These early-age effects of Mrp8/14 deficiency, in the absence of any external stressor, were unexpected. Taken together, our findings demonstrate a pro-steatosis rather than pro-inflammatory role of calprotectin within the aging liver. This appears to reflect a developmental-metabolic phenotype of Mrp14-KO mice that is manifest at a young age in the absence of pro-inflammatory stimuli.

## INTRODUCTION

Aging is associated with chronic low-grade inflammation and the accumulation of immune cell deposits within tissues. This process, termed “inflammaging”, may contribute to functional and cognitive decline with increased age, and is proposed to be an important target for development of drugs to promote healthy aging and increased healthspan [1]. The *Mrp8* (*S100a8*) and *Mrp14* (*S100a9*) genes encode pro-inflammatory proteins that together form the Mrp8-Mrp14 heterocomplex (calprotectin), which is abundant in myeloid cells as well as damaged or stressed tissues. *Mrp8* and *Mrp14* mRNAs have been used as *in situ* inflammation markers [2], but have also been identified as biomarkers of aging in mammalian tissues [3]. In humans, aging leads to increased *Mrp8* and *Mrp14* mRNA abundance in airway epithelia and throughout the central nervous system (temporal lobe, hippocampus, parietal lobe and frontal lobe) [3]. In mice, elevated *Mrp8* and *Mrp14* with aging is more widespread, occurring in skin, lung, liver, kidney, aorta, muscle, eye and central nervous system [3]. Increased *Mrp8* and *Mrp14* expression with age is likely due to immune cell infiltration into aging tissues, but may additionally reflect local responses of cells to age-related stress, damage or senescence [4, 5]. In either case, Mrp8/Mrp14 accumulation may contribute to chronic inflammation [6–9], enhance atherosclerosis and vascular disease [10], promote tumorigenesis [11], and facilitate plaque formation in the brain leading to memory impairment [12–15].

Nearly all functional studies of calprotectin have been performed using young mice [16–23]. The contributions of Mrp8 and Mrp14 to age-related pathology have therefore remained uncertain. *Mrp14*(−/−) mice lack *Mrp14* mRNA but are also deficient for the calprotectin complex due to instability of the Mrp8 protein in the absence of Mrp14 [16, 17]. Under normal physiological conditions, studies of young *Mrp14*(−/−) mice have frequently failed to identify phenotypes that differ significantly from wild-type controls [16, 17]. Phenotypic differences have emerged, however, following stressful or pro-inflammatory stimuli, such as *E. coli*-induced sepsis [18], intraperitoneal LPS administration [6], carbon tetrachloride (CCl_4_) toxicity [19], lung injury [20], and infection [21–23]. In general, inflammatory responses to these treatments are diminished in *Mrp14*(−/−) mice as compared to wild-type controls. This likely reflects the pro-inflammatory activities of calprotectin, which include the enhancement of cytokine release [7], transendothelial migration of leukocytes [8], toll-like receptor 4 activation [6], and RAGE activation (receptor of advanced glycation end products) [9]. It is unknown whether these effects of Mrp8/Mrp14 also amplify chronic low-grade inflammation in the context of aging. If so, this would suggest new options for treating inflammaging, since efficacious and tolerable drugs have been developed to block Mrp8/14-driven inflammation (i.e., ABR-215757) [24–26].

In this study, we used microarray analysis to characterize hepatic gene expression profiles of wild-type (WT) and *Mrp14*(−/−) (KO) mice at young (5 months) and old (24 months) ages. We chose liver as a model system to study inflammaging because (i) *Mrp8* and *Mrp14* mRNAs are robustly elevated in old *versus* young liver [3], (ii) stress-dependent differences in hepatic inflammation have been identified between WT and KO mice [18, 19], and (iii) hepatic inflammation is a robust feature of aging that has been well-characterized by previous work [27–29]. Our findings reveal unexpected gene expression differences between WT and KO mice at a young age (in the absence of physiological stress), and address the hypothesis that Mrp8 and Mrp14 accumulation promotes age-related inflammation.

## RESULTS

### Mrp8/Mrp14 deficiency does not prevent inflammaging (liver, lung and skin)

**Figure 1:**
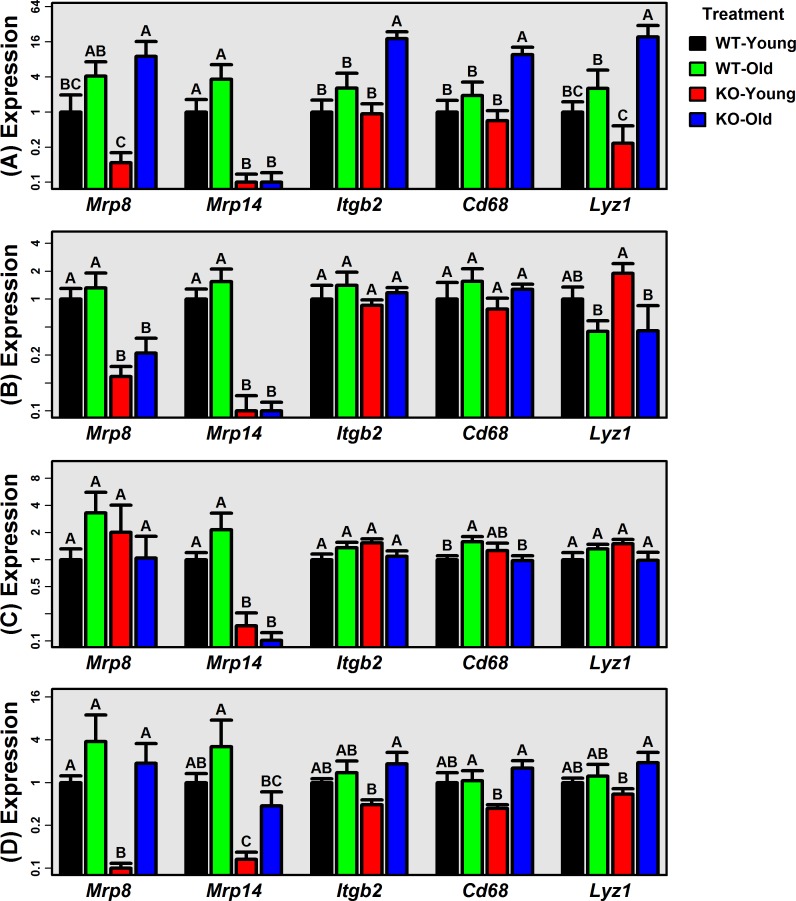
Expression of *Mrp8, Mrp14* and genes associated with age-related inflammation *(Itgb2, Cd68 and Lyz1)* RT-PCR was used to measure expression of *Mrp8*, *Mrp14*, *Itbg2*, *Cd68* and *Lyz1* in **A.** liver, **B.** lung, **C.** ear skin, and **D.** tail skin (WT and KO mice; young: 5 months; old: 24 months). Average relative expression is shown for each treatment (± 1 standard error; 5 ≤ *n* ≤ 9 per treatment), and expression of each gene is normalized to the average expression of the young-WT group. Expression of 18S ribosomal RNA (*Rn18s*) was used an endogenous control gene. Treatments that do not share the same letter differ significantly from one another (*P* < 0.05; Fisher's LSD).

Female WT and KO mice were maintained under pathogen free conditions for 5 or 24 months. Gene expression analysis of multiple organs was performed to assess *Mrp8*/*Mrp14* expression and evidence for tissue inflammation. As expected, *Mrp14* expression was significantly reduced in KO liver, lung, ear skin and tail skin (*P* < 0.05; Fisher's LSD; Figure 1). *Mrp8* expression was also significantly reduced in lung and tail skin from KO mice (*P* < 0.05; Fisher's LSD; Figure 1). In WT mice, *Mrp8* and *Mrp14* expression was always, on average, higher in old mice compared to young mice (Figure 1). These trends were marginally significant with respect to *Mrp8* expression in liver (FC = 4.13, *P* = 0.071), *Mrp8* expression in ear skin (FC = 3.32, *P* = 0.036), *Mrp14* expression in liver (FC = 3.64, *P* = 0.057) and *Mrp14* expression in ear skin (FC = 2.15, *P* = 0.064) (one-tail two-sample *t*-test).

We evaluated three gene expression markers of inflammation previously shown to be elevated with mouse aging (*Cd68*, *Itgb2* and *Lyz1*) [30]. We expected expression of these genes to be elevated with aging in WT mice only, but this was not the case (Figure 1). To the contrary, in liver and tail skin, *Cd68*, *Itgb2* and *Lyz1* expression was elevated with aging in KO mice only (*P* < 0.05; Fisher's LSD; Figure 1A). Inflammation-associated gene expression with aging was therefore not blunted by Mrp8/Mrp14 deficiency but was in fact enhanced in KO compared to WT mice.

### Hepatic gene expression profiles of young KO and WT mice differ under normal physiological conditions

We used Affymetrix Mouse Gene 2.1 ST microarrays to evaluate hepatic gene expression profiles of young WT (*n* = 5), young KO (*n* = 5), old WT (*n* = 6), and old KO (*n* = 8) mice. Unsupervised cluster analysis of expression profiles yielded partial separation of samples from each group (Figure S1A), which was more evident when samples were plotted with respect to principal components (Figure S1B). Within two-dimensional principal component space, KO and WT samples differed at a young age but then followed a similar aging trajectory, remaining distinct at 24 months of age (Figure S1B). Microarray estimates of *Mrp8* and *Mrp14* expression revealed expected trends, with increased *Mrp14* expression with aging in WT but not KO mice (Figure S1C).

Aging had significant effects on hepatic gene expression that were of similar magnitude in WT and KO mice. We identified 45 differentially expressed genes (DEGs) altered by aging in WT mice (11 age-increased; 34 age-decreased), along with 45 DEGs altered by aging in KO mice (24 age-increased and 21 age-decreased) (FDR < 0.10 with FC > 1.50 or FC < 0.67). We had expected stronger WT *vs*. KO expression differences at an older age, but this was not supported by differential expression analysis. We identified 215 DEGs from the comparison between young KO and WT mice (136 KO-increased; 79 KO-decreased), but comparison of old KO and WT mice yielded only 6 DEGs (KO increased: *Wdfy1*, *Rps3a1*; KO-decreased: *Mme*, *Fam167b*, *Aadac*, *Mrp14*) (FDR < 0.10 with FC > 1.50 or FC < 0.67). WT and KO genotypes thus showed greater differential expression at a young age. We did not identify genes with significant genotype-by-age interaction effects (FDR < 0.10); however, at a genome-wide level, old/young FCs were only modestly correlated between WT and KO mice (*r_s_* = 0.467; Figure S1D). Likewise, there was modest genome-wide correlation of KO/WT FCs between young and old mice (*r_s_* = 0.493; Figure S1E).

### Genes differentially expressed between WT and KO mice are associated with development, biosynthesis, lipid metabolism, and immunity

**Figure 2:**
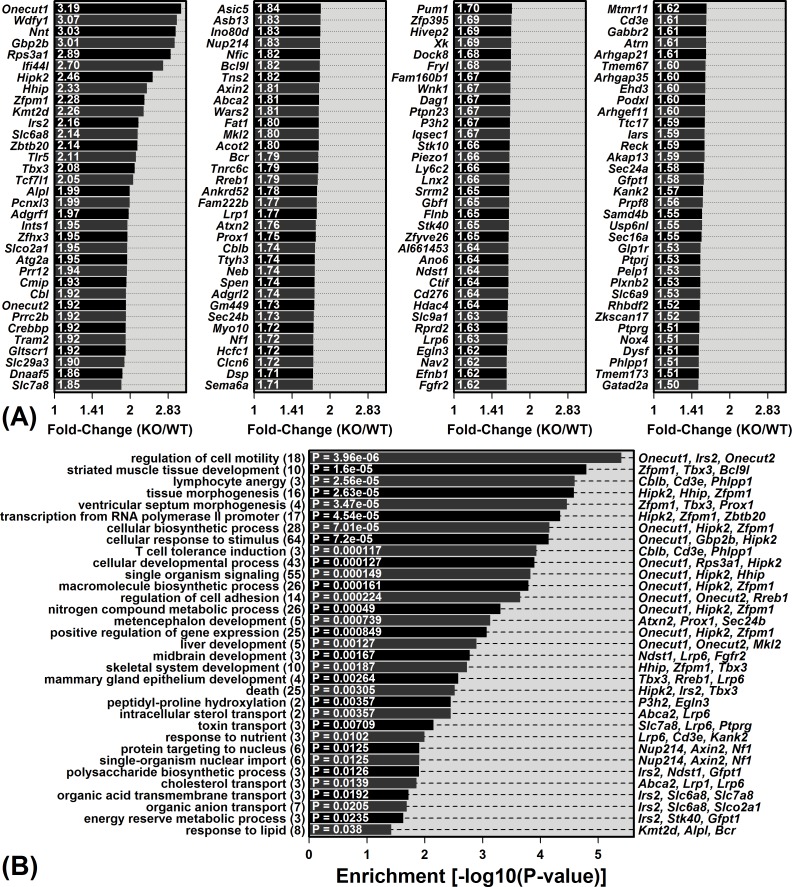
Genes with elevated expression in young KO mice compared to young WT mice and their associated gene ontology (GO) biological process (BP) terms **A.** The 136 DEGs with significantly elevated expression in young KO mice compared to young WT mice (FDR < 0.10 with KO/WT FC ≥ 1.50). **B.** GO BP terms most significantly enriched among the 136 DEGs. The number of DEGs associated with each GO BP term is listed in parentheses (left margin). DEGs most strongly elevated in KO mice are listed for each GO BP term (right margin).

**Figure 3:**
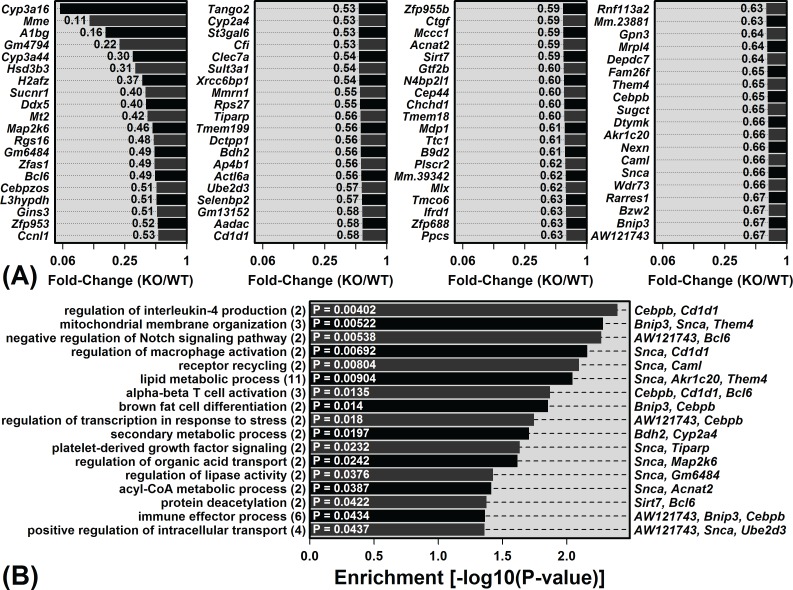
Genes with decreased expression in young KO mice compared to young WT mice and their associated gene ontology (GO) biological process (BP) terms **A.** The 79 DEGs with significantly decreased expression in young KO mice compared to young WT mice (FDR < 0.10 with KO/WT FC ≤ 0.67). **B.** GO BP terms most significantly enriched among the 79 DEGs. The number of DEGs associated with each GO BP term is listed in parentheses (left margin). DEGs most strongly decreased in KO mice are listed for each GO BP term (right margin).

*Mrp8* and *Mrp14* are expressed at low levels in livers from young WT mice (Figure S1C). We were therefore surprised to have identified a large number of DEGs (215) from the comparison between young WT and KO mice. Among the 136 DEGs with elevated expression in young KO mice, the most strongly elevated were *Onecut1*, *Wdfy1* and *Nnt* (Figure 2A). The complete set of 136 KO-increased DEGs showed significant enrichment for diverse gene ontology (GO) biological process (BP) terms, including liver development, cellular biosynthetic process, response to lipid, and lymphocyte anergy (*P* < 0.05; Figure 2B). Of 79 DEGs with decreased expression in young KO mice, the most strongly decreased included *Cyp3a16*, *Mme* and *A1bg* (Figure 3A). These 79 DEGs were also significantly enriched with respect to diverse GO BP terms, including lipid metabolic process, alpha-beta T-cell activation and immune effector process (*P* < 0.05; Figure 3B).

### Genes differentially impacted by aging in WT and KO mice are associated with ribosome biogenesis and metabolic processes

Most genes were similarly altered by aging in WT and KO mice (Figure S1D). We did not detect significant genotype-by-age interaction effects after adjustment for multiple comparisons (FDR < 0.10), although we did identify 729 genes with genotype-by-age interaction effects based upon a less conservative comparison-wise significance threshold (*P* < 0.05). Examples of genes with age-decreased expression in WT mice but age-increased expression in KO mice included *Srfbp1*, *Ccnl1* and *Chd1l* (Figures S2A, S2C and S2D). Genes following this pattern were enriched with respect to GO BP terms associated with ribosome biogenesis and the metabolism of RNA, linoleic acid, heterocycles, aromatic compounds, organic cyclic compounds, drugs and icosanoids (*P* < 0.05). Conversely, examples of genes with age-increased expression in WT mice but age-decreased expression in KO mice included *Mrps36*, *Dnlz*, and *Paqr6* (Figures S2B, S2E and S2F). Genes exhibiting this pattern were significantly enriched with respect to GO BP terms corresponding to long-chain fatty acid metabolism and positive regulation of cell division (*P* < 0.05).

### Genes specifically expressed by B-cells and T-cells are more strongly elevated with aging in KO mice as compared to WT mice

Calprotectin is a leukocyte chemoattractant with multiple other pro-inflammatory functions [6–9]. We therefore expected that WT and KO mice would differ with regard to hepatic immune cell composition, particularly following the onset of age-related inflammation. To address this possibility, data from the Immunological Genome Project (IGP) [31] was used to identify 100 “signature genes” specifically expressed by each of 222 immune cell populations (C57BL/6 mice; see Methods). We then assessed whether signature genes for any one cell population were systemically altered in our treatment comparisons [32].

**Figure 4:**
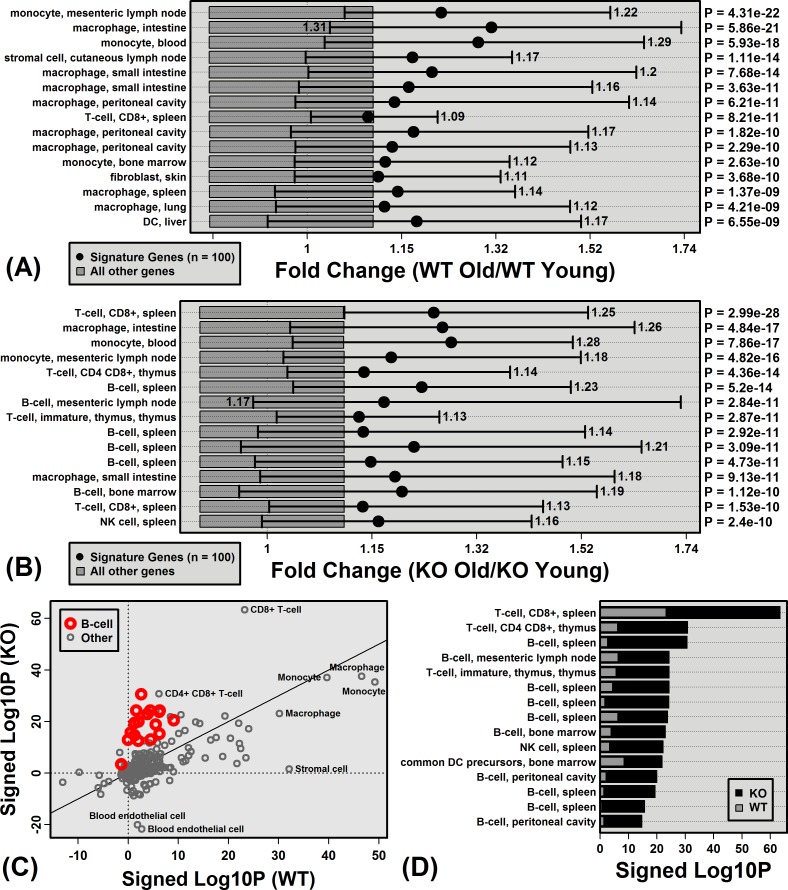
Aging increases hepatic expression of genes specifically expressed by monocytes, macrophages, B-cells and T-cells Immunological Genome Project (IGP) cell populations with signature genes biased toward age-increased expression in **A.** WT mice and **B.** KO mice. Black dots represent the average old/young FC of signature genes, with bars spanning the middle 50% of FC estimates among signature genes (i.e., 25th to 75th percentiles). Dark grey boxes outline the middle 50% of FC estimates among non-signature genes. P-values for each cell population were obtained by comparing FC estimates between signature and non-signature genes (right margin; Wilcoxon rank sum test). Part **C.** compares p-values obtained from all 222 cell populations for KO and WT mice. P-values were log_10_-transformed (Log10P) and signed to indicate whether signature genes are biased towards age-decreased expression (Lop10*P* < 0) or age-increased expression (Log10*P* > 0). Part **D.** lists cell populations for which Log10P is higher in KO *versus* WT mice.

Genes specifically expressed by immune cell populations were biased towards age-increased expression in both WT and KO mice, consistent with age-related hepatic inflammation (Figure 4). In WT mice, monocyte and macrophage signature genes were the most strongly elevated with aging (compared to genes not specifically expressed by these cell types; Figure 4A). In KO mice, monocyte/macrophage signatures genes also showed increased expression with age, although additionally genes specifically expressed by T-cells and B-cells were biased toward age-increased expression (Figure 4B). Among the 222 cell populations, B-cell and T-cell signatures genes were the most differentially altered by aging in KO and WT mice, with genes specifically expressed by these cell types elevated more consistently in KO compared to WT mice (Figure 4C and 4D).

**Figure 5:**
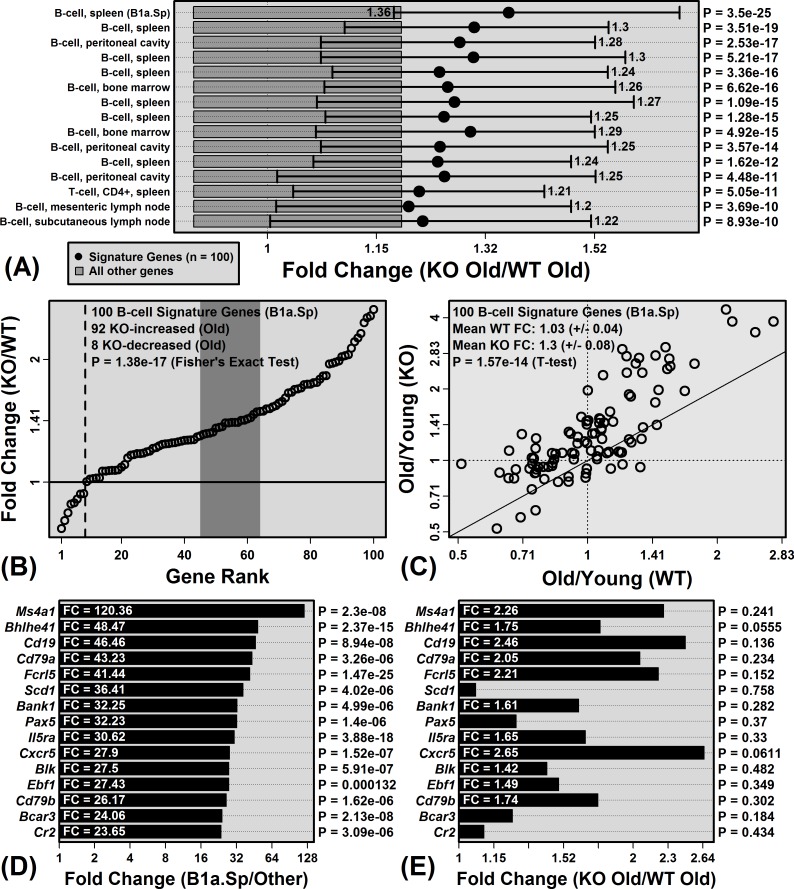
Genes specifically expressed by B-cells have elevated expression in old KO mice compared to old WT mice **A.** Immunological genome project (IGP) cell populations with signature genes biased toward elevated expression in old KO mice compared to old WT mice. Black dots represent the average signature gene FC between old KO and old WT mice, with bars spanning the middle 50% of FC estimates among signature genes (i.e., 25th to 75th percentiles). Dark grey boxes outline the middle 50% of FC estimates among non-signature genes. P-values for each cell population were obtained by comparing FC estimates between signature and non-signature genes (right margin; Wilcoxon rank sum test). **B.** FC estimates (old KO/old WT) for the 100 signature genes associated with the top-ranked B-cell population from part **A.** (B1a.Sp). The dotted vertical line indicates the number of KO-decreased genes. The dark grey region is the middle 95% of the null distribution for this value (Fisher's Exact Test). **C.** Comparison of FC estimates (old/young) between KO and WT mice for the 100 signature genes associated with B1a.Sp. **D.** Genes most highly expressed by B1a.Sp relative to the other 221 IGP cell populations. The chart shows the FC and p-value obtained from the comparison between B1a.Sp and the 221 other IGP cell populations. **E.** FC estimates (old KO/old WT) for genes shown in **D.** and their associated *p*-values (right margin).

The unique B-cell signature gene pattern was more apparent from the comparison between old KO and old WT mice (Figure 5). Of cell populations with signature genes most strongly biased towards KO-increased expression at an older age, 14 of the top-ranked 15 were B-cell populations (Figure 5A). The trend was strongest for IGP cell population B1a.Sp (*P* = 3.5 × 10^−25^), which is a spleen-derived B-cell subset characterized by expression of IgD, IgM, CD45R, CD24, CD19 and CD43 (negative for expression of AA4.1, CD23, and CD21/35). Of 100 genes most specifically expressed by this cell population, 92 showed higher expression on average in old KO mice compared to old WT mice (*P* = 1.38 × 10^−17^; Fisher's Exact test; Figure 5B). Among these 100 genes, moreover, the average old/young FC in WT mice was 1.03 (± 0.04), whereas the average old/young FC in KO mice was 1.30 (± 0.08) (*P* = 1.57 × 10^−14^; two-sample *t*-test). Each of the 15 genes most specifically expressed by B1a.Sp was expressed more highly on average in old KO mice compared to old WT mice (e.g., *Ms4a1*, *Bhlhe41*, *Cd19*; Figure 5D and 5E). Consistent with these observations, CD45R (B220) staining intensity was stronger in hepatic tissue from old KO mice as compared to old WT mice (Figure S3).

### Mrp8/Mrp14 deficiency blunts progression of age-related steatosis

Genes with significantly decreased expression in young KO compared to young WT mice were disproportionately associated with lipid metabolism (e.g., *Snca*, *Akr1c20*, *Them4*; Figure 3B). This prompted us to ask whether development of steatosis with aging is diminished in KO mice [33]. A microarray dataset (GSE46209) [34] was therefore used to identify genes with significantly greater expression in intra-abdominal white adipose tissue (WAT; *n* = 3) compared to liver (*n* = 3) (C57BL/6 mice; age 6-8 weeks). A subset of 100 genes with significantly elevated expression in WAT (FDR < 0.10; FC > 1.50) was then analyzed further as WAT signature genes.

**Figure 6:**
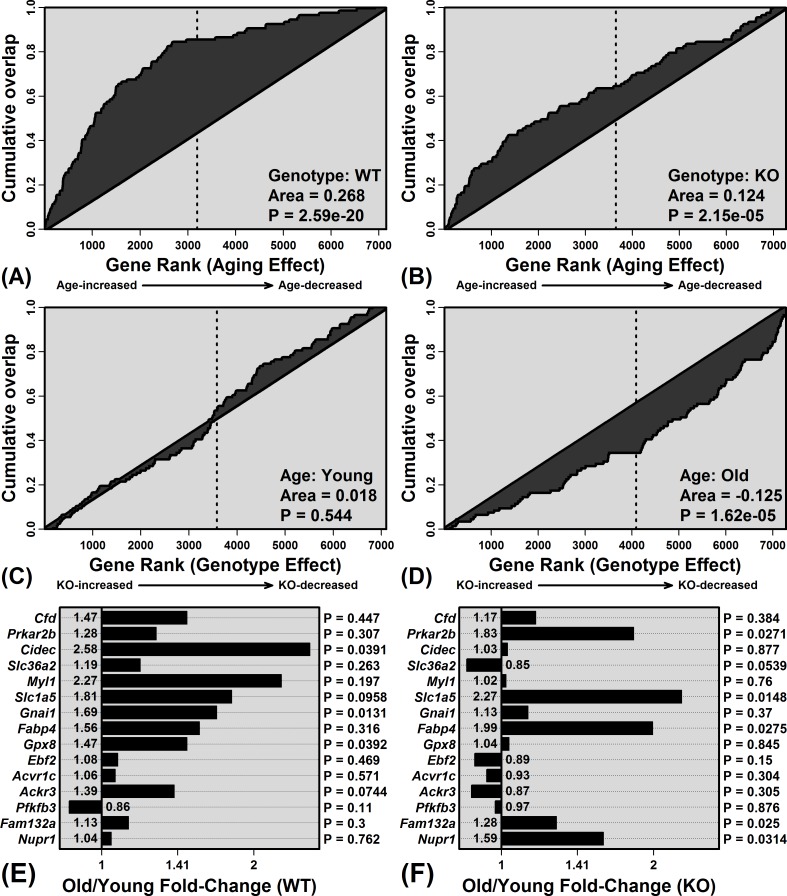
Elevated expression of white adipose tissue (WAT) signature genes with aging is blunted in KO compared to WT mice Enrichment of WAT signature genes among genes with age-increased expression in **A.** WT and **B.** KO mice. Parts **A.** and **B.** show cumulative overlap of the 100 WAT signature genes relative to a list of genes ranked according to how strongly their expression is increased by aging (age-increased: left of vertical line; age-decreased: right of vertical line). Significant overlap of WAT signature genes and age-increased genes is indicated by a positive area between the cumulative overlap curve and diagonal. Similarly, **C.** and **D.** show cumulative overlap between WAT signature genes and genes ranked according to how strongly their expression is elevated in KO compared to WT mice (C: young mice; D: old mice; KO-increased: left of vertical line; KO-decreased: right of vertical line). Parts **E.** and **F.** list WAT signature genes and their FC estimates (old/young) in **E.** WT and **F.** KO mice. The 15 WAT signature genes with highest FC (WAT/liver) are listed.

**Figure 7:**
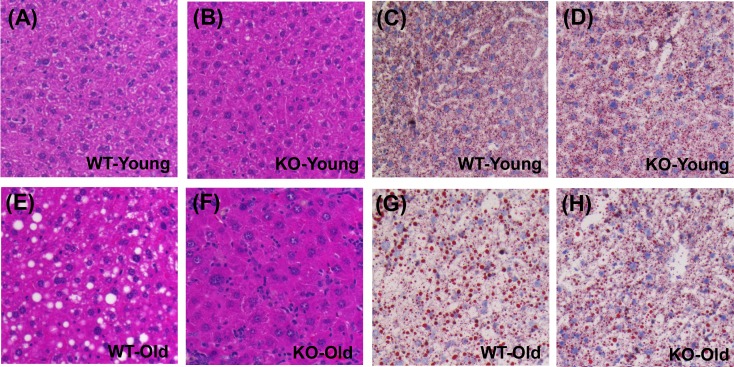
Hepatocellular vacuolation and oil red O staining is attenuated in livers from old KO compared to old WT mice **A.**, **B.**, **E.**, **F.** Hematoxylin and eosin **H.**&**E.** stain in young and old WT/KO mice. **C.**, **D.**, **G.**, **H.** Oil red O staining for lipid droplets in young and old WT/KO mice.

The 100 WAT signature genes were disproportionately elevated with aging in WT mice (*P* = 2.59 × 10^−20^; Figure 6A), consistent with age-related steatosis [33]. WAT-specific genes were also elevated with aging in KO mice, but this trend was weaker compared to WT mice (*P* = 2.15 × 10^−5^; Figure 6B). Of the 15 genes most specifically expressed by WAT, average expression of 14/15 was elevated with aging in WT mice, and fewer (10/15) were similarly elevated in KO mice (Figure 6E and 6F). WAT signature genes were also biased toward decreased expression in old KO mice as compared to old WT mice (*P* = 1.62 × 10^−5^; Figure 6C). Consistent with these findings, histological analyses revealed hepatocellular vacuolation in old WT mice, along with increased frequency of lipid droplets identified by oil red O staining (Figure 7).

These trends suggested that KO mice are partially resistant to age-related steatosis. We thus predicted that hepatic expression differences between old KO and old WT mice would parallel those of treatments that attenuate steatosis. One such treatment is SRT1720, which is a Sirt1 activator previously shown to block steatosis in fasting mice [35]. A microarray dataset (GSE19102) was therefore used to identify 100 genes decreased by SRT1720 in mouse liver [36] (C57BL/6 strain; see Methods). We confirmed that these genes were disproportionately elevated with aging in WT mice (*P* = 4.95 × 10^−11^; Figure S4A). As predicted, however, the strength of this trend was diminished in KO mice (*P* = 7.03 × 10^−3^; Figure S4B), and fewer of the most strongly SRT1720-decreased genes were elevated with aging in the KO genotype (Figures S4E and S4F). The 100 SRT1720-decreased genes were also disproportionately reduced in old KO mice compared to old WT mice (*P* = 0.003; Figure S4D). Gene expression shifts in Mrp8/Mrp14-deficient mice thus paralleled hepatic responses to the anti-steatosis agent SRT1720.

## DISCUSSION

Inflammation is a robust feature of aging and may facilitate development of chronic diseases to limit functional capacity [1]. Mrp8 and Mrp14 (calprotectin) have been identified as biomarkers of the aging process [3], but while their pro-inflammatory activity has been documented in young mice under acute stress [16–23], their contributions to chronic inflammation at older ages remains unclear. This study compared Mrp14-KO and WT mice at young and old ages but obtained no evidence that age-related inflammation is blunted by Mrp8/Mrp14 deficiency. Old KO mice in fact showed stronger elevation of B-cell and T-cell-associated gene expression, suggesting that calprotectin may protect against age-related inflammation in WT mice. Unexpectedly, however, KO mice were deficient for expression of lipid metabolism genes at an early age, and subsequently exhibited less progressed steatosis in old age. These findings indicate that, while Mrp8/Mrp14 provide biomarkers of age-related inflammation [2, 3], their activity within aging tissues may not be pro-inflammatory as observed in young mice following acute stress. However, our findings show that Mrp14-KO mice can provide a model system for studies of immune-hepatocyte interactions and their influence on lipid metabolism and steatosis.

Mouse aging frequently leads to increased expression of mRNAs expressed by immune cells that become fixated within non-myeloid tissues during the course of aging [27, 37]. This has frequently been observed in microarray comparisons between young and old mouse tissues, which have revealed elevated expression of immune cell-derived proteins, such as Mrp8 and Mrp14 [29, 30, 37–40]. We hypothesized that elevated Mrp8/Mrp14 expression with aging drives accumulation of immune cells within aging tissues. Surprisingly, however, Mrp14-KO mice did not appear resistant to age-related inflammation, and in contrast, KOs showed stronger elevation of B-cell and T-cell-associated transcripts with liver aging (Figures 4 and 5). It is possible that this result was due to compensatory responses that occur in Mrp14-KO mice, given the absence of functional calprotectin, which enable development of age-associated inflammation *via* unconventional pathways. Our microarray analysis, however, did not suggest strong compensatory shifts related to inflammatory gene expression, and to the contrary, genes associated with “lymphocyte anergy” were enriched among KO-increased DEGs (Figure 2). Alternatively, our findings may reflect a more complex role of Mrp8/14 at an old age, which is characterized by low-grade chronic (non-acute) inflammation combined with elevated oxidative stress. Such conditions may influence Mrp8/14 function [41, 42]. For instance, Mrp8/14 is recognized as a neutrophil chemoattractant [21], but oxidized forms of human S100A8/A9 repel neutrophils [41, 42]. Consistent with this activity, Mrp14-KO mice exhibited stronger dermal T-cell recruitment following treatment with the oxidative stress agent 7,12-dimethylbenz(a)anthracene (DMBA) [43]. Finally, it is possible that Mrp8/14 is responsive to age-related inflammation, but does not itself serve as a causal trigger. Other events or factors with a leading role, for instance, might include progressive tissue damage or accumulation of auto-antibodies [44].

Most studies have identified Mrp14-KO phenotypes only in the context of acute pro-inflammatory stimuli [16–23], which has fostered the view that KO and WT mice differ little under standard physiological conditions [16, 17]. This conflicts with our observations, however, since we identified 215 genes differentially expressed between livers from KO and WT mice at 5 months of age, in the absence of any stressful or pro-inflammatory conditions (Figures 2 and 3). Most of these genes were not directly associated with immune response, but were associated with “housekeeping” cellular processes, such as cell motility, development, transport and metabolism (Figures 2 and 3). These observations may reflect involvement of Mrp8/14 in processes instrumental for normal liver development. The developmental role of Mrp8, for instance, is established from the fact that Mrp8-KO embryos do not survive gestation and are resorbed, apparently due to maternal rejection [45]. During development, expression of Mrp8/14 is mostly restricted to the myeloid lineage, but expression is detected in liver starting at day 11 of gestation concurrent with myelopoiesis [46]. Potentially, therefore, absence of Mrp14 expression in KO mice influences hepatic development to account for the gene expression phenotype we observed at 5 months of age. Alternatively, Mrp8 and Mrp14 have been associated with intracellular processes that may be important for (post-natal) functioning of hepatocytes or Kupffer cells (e.g., calcium sensing and homeostasis, protein phosphorylation, NADPH oxidase activation, transport, or cytoskeleton regulation) [11, 47, 48]. Either developmental or intracellular functions of Mrp8/14, therefore, may explain hepatic gene expression differences between WT and KO mice at young ages under normal conditions. Such baseline genotype differences may be important to consider in studies that compare acute inflammatory responses of Mrp14-KO and WT mice [18, 19].

Plasma calprotectin has been identified as a marker of obesity and may mediate obesity-associated diseases such as atherosclerosis [10, 49]. Increased obesity with aging is common along with elevated fat to lean mass ratio, partly due to declines in circulating insulin-like growth factors [50]. These effects are frequently associated with accumulation of fat deposits within non-adipose tissues, including the heart, muscle and liver [51]. Within the liver, such fat deposits can be a first step leading to non-alcoholic fatty liver disease (NAFLD), steatohepatitis, cirrhosis, and potentially, hepatocellular carcinoma (HCC) [51]. In our study, Mrp14-KO mice showed resistance to age-related intrahepatic fat accumulation, suggesting a potentially favorable effect of Mrp8/14 deficiency on aging trajectory. This may be related to the intrinsic capacity of calprotectin to bind and transport fatty acids within hepatocytes or associated immune cell populations [52]. Any anergic effect of Mrp14-KO on Kupffer cells, in particular, may influence steatosis, since Kupffer cell depletion was previously shown to attenuate steatosis in rodents [53]. Within aging liver, Kupffer cells or other immune populations may also release pro-steatosis cytokines, such as IL-1β, TNF-α and IL-22 [54, 55], which disrupt bile acid synthesis and cholesterol efflux to promote cholesterol accumulation [55, 56]. Recently, moreover, transgenic mice overexpressing human *S100A8*/*A9* (*Mrp8*/*14*) and *S100A12* were demonstrated to have elevated lipid content in peritoneal macrophages, potentially due to RAGE-dependent induction of IL-22 expression [55]. Expression of these cytokines (IL-1β, TNF-α, IL-22) may be positively regulated by Mrp8/14 [57, 58]. Attenuation of steatosis in Mrp14-KO mice, therefore, may be explained by several potential mechanisms, including altered lipid binding and metabolism, diminished Kupffer cell activity, or decreased expression of cytokines that control lipid storage and processing.

Activation of inflammatory pathways is closely associated with increasing chronological age, but “healthy aging” in humans seems to involve dampening of these responses [59]. Our findings emphasize the complexity of circuits involved in such pathways, in which archetypal pro-inflammatory proteins such as Mrp8 and Mrp14 can have multiple roles, which include immune response but extend from development to lipid metabolism. Mrp14-KO mice provide a model system for understanding interactions among these systems. In future studies, therefore, it will be valuable to establish whether our observations extend to both sexes, to identify B and T cell subpopulations infiltrating hepatic tissue in old KO mice, and to characterize signals and homing mechanisms that mediate this process. Finally, it is interesting to note that resistance to steatosis has often been linked to interventions with broader impacts on aging, such as SRT1720 [35], resveratrol [60], rapamycin [61], and metformin [62]. Potentially, therefore, further studies of Mrp14-KO mice will uncover age-specific phenotypes beyond those identified in the current study, providing further opportunity to investigate immune-adipose interactions and their connections to development and aging outcomes.

## MATERIALS AND METHODS

### Animal husbandry

Female *Mrp14*(−/−) (KO) and control (WT) mice with C57BL/6 background were maintained from birth until 5 or 24 months within the Case Western Reserve University (CWRU) animal vivarium. The age of 5 months was chosen to ensure that sexual maturation was complete in mice from the young group [63]. The age of 24 months was chosen because mice older than this age may be experiencing end-stage disease processes, which would itself have physiological effects that could obscure processes related to basic aging mechanisms [63]. Mice were maintained under specific-pathogen free conditions and provided standard rodent chow diet (Prolab^®^ Isopro^®^ RMH 3000 5P76; 22% protein, 5% fat). Mice were fasted 4 hours prior to euthanasia but provided access to water at all times. Sacrifice was performed by cervical dislocation following injection with euthanasia solution (ketamine, 16.5mg/mL; xylazine, 1.65 mg/mL). Both WT and KO mice appeared healthy at 24 months of age without macroscopic evidence of pathology. Tissues were harvested from mice upon sacrifice, flash-frozen in liquid nitrogen, and placed at −80°C until further processing. All protocols were consistent with guidelines issued by the American Association for Accreditation of Laboratory Animal Care and approved by the CWRU Institutional Animal Care and Use Committee (Case Western Reserve University, Cleveland, OH).

### Microarray analysis of hepatic gene expression

Genome-wide expression of 24 liver samples was evaluated using Affymetrix Mouse Gene 2.1 ST microarrays (young-WT: *n* = 5; young-KO: *n* = 5; old-WT: *n* = 6; old-KO: *n* = 8). Raw and normalized data have been deposited in the Gene Expression Omnibus (GEO) database (http://www.ncbi.nlm.nih.gov/geo/query/acc.cgi?token=gvmdcywkthkpbct&acc=GSE79182) [64]. Flash-frozen samples were pulverized and extraction of total RNA was performed using the RNeasy mini kit (Qiagen, cat no. 74104) with on-column DNase digestion (Qiagen, cat. no. 79254). RNA concentrations were measured using a NanoDrop spectrophotometer and RNA integrity was evaluated using the Agilent Bioanalyzer. Microarray processing was carried out with biotinylated cDNA prepared according to the Affymetrix Plus WT kit protocol (GeneChip^®^ WT Plus Reagent Kit Manual P/N 703174 Rev. 2). After labeling, 2.53 μg of cDNA was hybridized at 48°C on Mouse Gene ST 2.1 arrays, which were then washed, stained and scanned using the Affymetrix GeneTitan system (software version 3.2.4.1515).

Quality control (QC) inspection of raw CEL files was performed using NUSE (normalized unscaled standard errors) and RLE (relative log expression) probe-level metrics [65]. Median NUSE and RLE values were consistent across arrays and therefore all 24 samples were retained for further statistical analysis. The robust multichip average algorithm (RMA) was used to perform background subtraction, quantile normalization and expression intensity summarization (R package: oligo; function: rma) [66, 67]. RMA normalization was performed at the gene level using the “target = core” option for the rma function [67] (R package: oligo). This yielded expression intensity estimates for 19513 protein-coding human genes. Genes with below-background expression were excluded from differential expression analyses (see below). A gene was considered to have above-background expression if the expression intensity of at least one associated probe set was significantly above-background (*P* < 0.05; Wilcoxon signed rank test) [68]. This was evaluated for each probe set based upon comparison of signal intensities between perfect match (PM) and mismatch (MM) probes (R package: oligo; function: paCalls with method = “PSDABG”) [67]. Given this criterion, between 12525 and 15555 genes per sample were identified as having above-background expression (14450 genes on average among the 24 samples).

Differential expression analysis was performed using linear modeling with moderated t-statistics (R package: limma; functions: lmFit and eBayes) [69]. Differentially expressed genes (DEGs) were identified with respect to four comparisons (WT-young *vs*. KO-young; WT-old *vs*. KO-old; WT-old *vs*. WT-young; KO-old *vs*. KO-young). We also evaluated the significance of genotype × age interaction effects to identify genes for which expression is differentially impacted by aging in WT and KO genotypes [70]. Differential expression analysis was performed only for genes with above-background expression with respect to at least one-third of the samples involved in each comparison. Given this criterion, between 15143 and 15647 genes were included in differential expression analyses for comparisons listed above. Raw p-values were calculated for each included gene and adjusted using the Benjamini-Hochberg method to control the false discovery rate [71]. Unless otherwise stated, DEGs were identified based upon an FDR threshold of 0.10 with estimated FC greater than 1.50 or less than 0.67. A conditional hypergeometric test was used to identify GO BP terms enriched among DEGs relative to a background set of liver-expressed protein-coding genes [72]. Rank-based gene set enrichment analysis (GSEA) was performed by comparing foreground and background gene sets using detection rate curves and the area under the curve (AUC) metric proportional to the Wilcoxon-Mann-Whitney statistic [73].

### Immunological genome project (IGP)

Gene expression data from the Immunological Genome Project (IGP) was used to identify “signature genes” specifically expressed by 222 immune cell populations [31, 32]. Raw CEL files associated with the IGP project (Phase I) were downloaded from GEO under accession number GSE15907 (Affymetrix Mouse Gene 1.0 ST Array). The complete dataset included 682 data samples corresponding to 222 different cell populations (i.e., approximately 3 replicates per cell population; male C57BL/6 mice). RMA normalization was performed as described above using the “target = core” option for gene level estimation of expression intensities on the log_2_ scale [67] (R package: oligo). Differential expression analysis was performed for each cell population by comparing expression between samples for that cell population and all other IGP samples included in the dataset. These calculations were performed using linear modeling with moderated t-statistics as described above (R package: limma; functions: lmFit and eBayes) [69]. These comparisons detected an average of 296 DEGs with significantly elevated expression (FDR < 0.10 with FC > 1.50) among the 222 cell populations (minimum: 2 DEGs; maximum: 1430 DEGs; median: 185 DEGs). However, to equalize cell populations with regard to the number of signature genes analyzed, we considered the 100 genes most highly expressed by each cell population relative to other cell types. For any given cell population, this was done by first selecting genes with elevated expression in that cell type (FC > 1), then selecting the 150 of these genes with lowest p-value, and finally, selecting the 100 of these genes with highest estimated FC. The same number of signature genes (100) was therefore selected for each cell population based upon p-value and FC criteria.

### Additional microarray datasets

Adipose and an independent set of liver microarray samples were not included within the IGP dataset for evaluation of gene expression patterns potentially indicative of steatosis. To identify adipose signature genes, therefore, we analyzed a microarray experiment that included samples from intra-abdominal WAT (*n* = 3) and liver (*n* = 3) (GSE46209) [34] (Affymetrix Mouse Genome 430 2.0 array; C57BL/6 mice; age 6-8 weeks). Expression intensities were calculated using robust multichip average (RMA) and differential expression between WAT (GSM1126365, GSM1126366, GSM1126367) and liver (GSM1126359, GSM1126360, GSM1126361) samples was evaluated using moderated t-statistics [66, 69]. This comparison yielded 1591 DEGs with significantly elevated expression in WAT compared to normal liver (FDR < 0.10 with FC > 1.50). Of these, we selected 100 as WAT signature genes using the FC/p-value ranking procedure described above for IGP cell populations.

Genes negatively regulated by SRT1720 were identified by comparing gene expression in livers from mice provided high fat diet with SRT1720 (100 mg/kg for 26 weeks) (GSM473339, GSM473340, GSM473341) to livers from mice provided only a high fat diet (GSM473333, GSM473334, GSM473335) (male C57BL/6J mice). Differential expression analysis was performed using moderated t-statistics as described above [69]. This comparison yielded only 12 DEGs with significantly decreased expression in SRT1720-fed mice (FDR < 0.10 with FC < 0.67) although 125 DEGs were identified based on less conservative criteria (FDR < 0.10 with FC < 1.00). Consistent with above analyses, we analyzed a subset of 100 SRT1720-decreased DEGs, by first identifying the 150 SRT1720-decreased DEGs with lowest p-value, and subsequently selecting the 100 of these with lowest FC (SRT1720-treated/control).

### Real time quantitative reverse transcription PCR (RT-PCR)

Pre-designed TaqMan^®^ primer assays were purchased from Applied Biosystems (*Mrp8*, cat. no. Mm00496696_g1; *Mrp14*, cat. no. Mm00656925_m1; *Cd68*, cat. no. Mm03047343_m1; *Itgb2*, cat. no. Mm00434513_m1; *Lyz1*, cat. no. Mm00657323_m1). RT-PCR reactions were performed using the 7900HT Fast Real-time PCR system with Fast 96-Well Block Module (Applied Biosystems, cat. no. 4351405). Cycle threshold values of target genes from each sample were normalized using 18S ribosomal RNA (*Rn18s*) as an endogenous control gene (Applied Biosystems, cat. no. Mm04277571_s1). Differential expression analysis and estimation of FC between treatment groups was then performed using the 2^−ΔΔCt^ method [74]. RT-PCR analysis with respect to liver samples yielded fold-change estimates consistent with those generated by microarray analysis (*Mrp8*, *Mrp14*, *Cd68*, *Itgb2* and *Lyz1*).

### Immunohistochemistry

Flash frozen hepatic tissue samples were processed for staining with Hematoxylin and Eosin (Anatech LTD, Cat. No. 832 and 842) or Oil Red O (Rowley Biochemical, Cat. No. #H-503-1B). Immunohistochemical stains for CD45R (B220) were performed using a working concentration of 10 μg/mL (Biolegend, Cat. No. 103239).

## SUPPLEMENTARY MATERIAL FIGURES


