# Supplementary material for: Deficiency of myeloid-related proteins 8 and 14 (Mrp8/Mrp14) does not block inflammaging but prevents steatosis
Source: Oncotarget. 2016 May 21;7(24):35535–51. doi: 10.18632/oncotarget.9550 (PMC5094943; doi:10.18632/oncotarget.9550)
Supplement: Supplementary file 1 [file oncotarget-07-35535-s001.pdf]

Deficiency of myeloid-related proteins 8 and 14 (Mrp8/Mrp14) does not block inflammaging but prevents steatosis

Supplementary Material

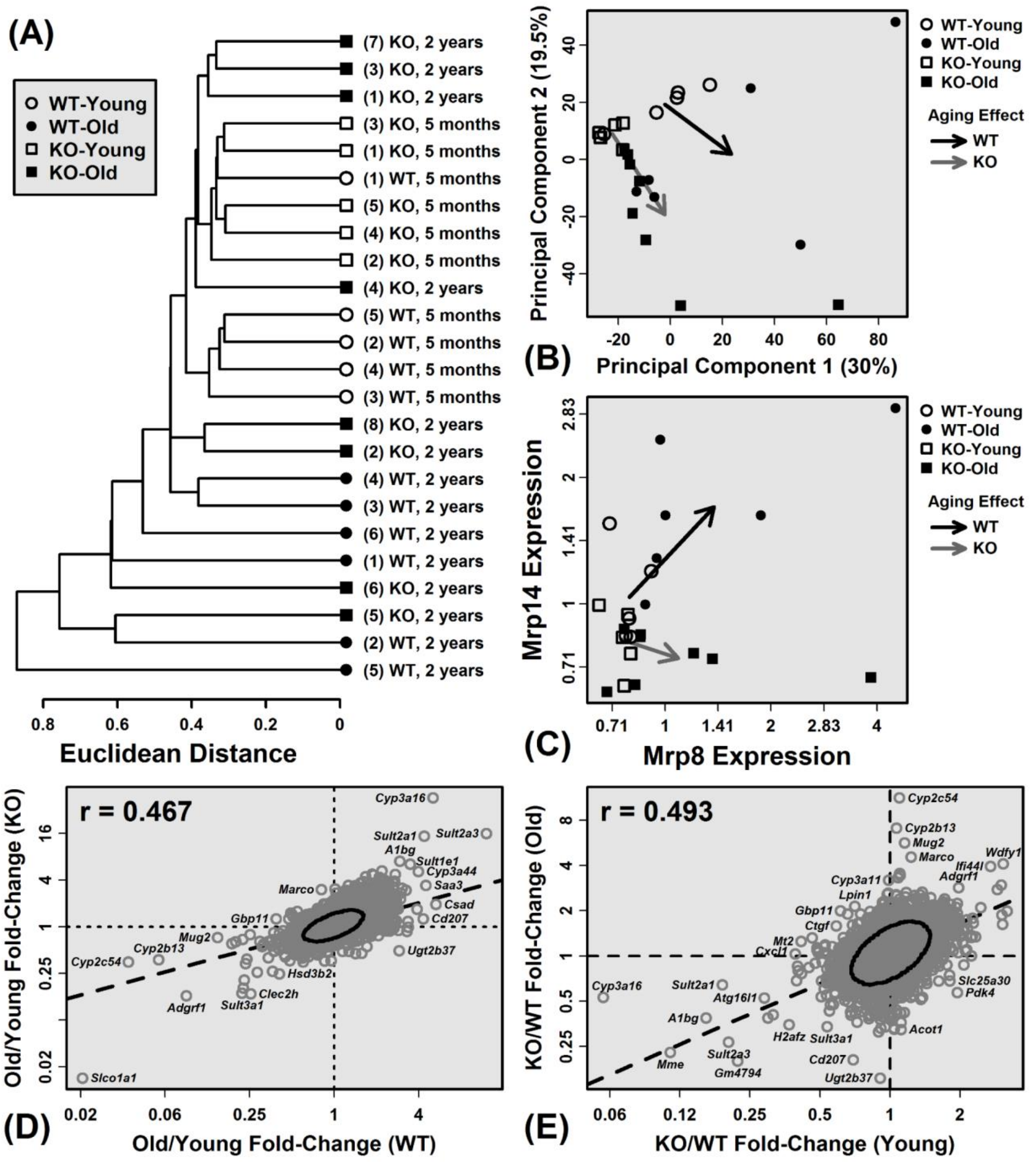

Figure S1. Hepatic gene expression profiling of WT and KO mice. (A) Hierarchical cluster analysis of gene expression profiles. (B) Principal components analysis. Samples are plotted with respect to the

first two principal components (calculated from the expression of protein-coding genes). The aging trajectory for each genotype, respectively, is illustrated by arrows connecting the bivariate mean of young samples to the bivariate mean of old samples. (C) Microarray expression estimates of *Mrp8* and *Mrp14*. Arrows illustrate the aging trajectory for each genotype, respectively (i.e., young bivariate mean → old bivariate mean). (D) Genome-wide correlation between FC estimates (old/young) calculated for WT (horizontal axis) and KO (vertical axis) genotypes. (E) Genome-wide correlation between FC estimates (KO/WT) calculated for young (horizontal axis) and old (vertical axis) mice. In (D) and (E), genes representing bivariate outliers are labeled and the ellipse outlines the 90% of protein-coding genes closest to the bivariate mean (Mahalanobis distance).

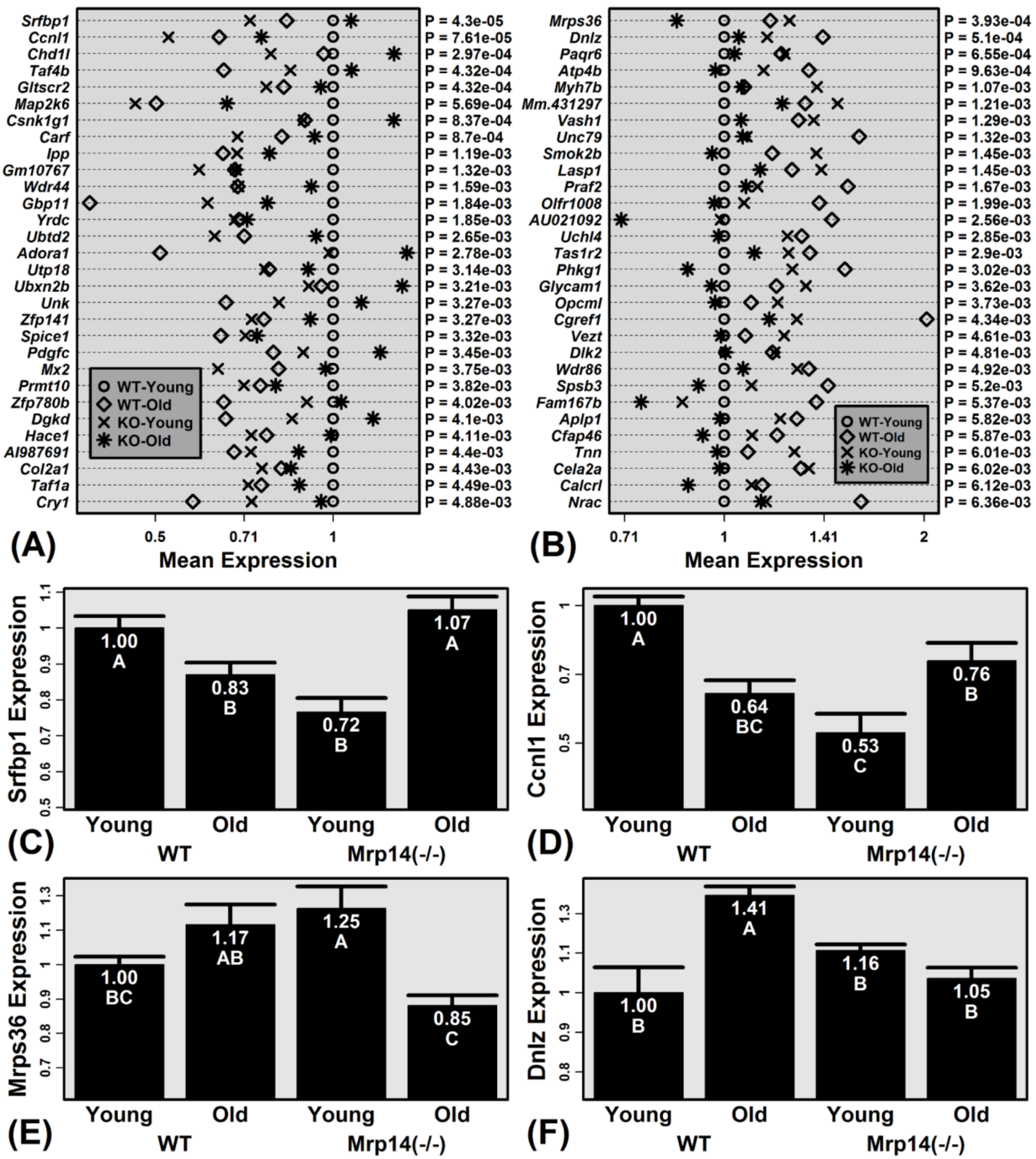

Figure S2. Genes differentially impacted by aging in WT and KO mice. (A) Genes decreased by aging in WT mice but increased by aging in KO mice ($P < 0.05$; genotype × age interaction effect). (B) Genes increased by aging in WT mice but decreased by aging in KO mice ($P < 0.05$; genotype × age interaction effect). Parts (C) – (F) show average expression of exemplar genes. Treatments with different letters differ significantly from one another ($P < 0.05$; Fisher's LSD). In (A) – (F), expression is normalized to the average expression of the young WT group. In (A) and (B), p-values from the linear model test for genotype × age interaction are listed (right margin).

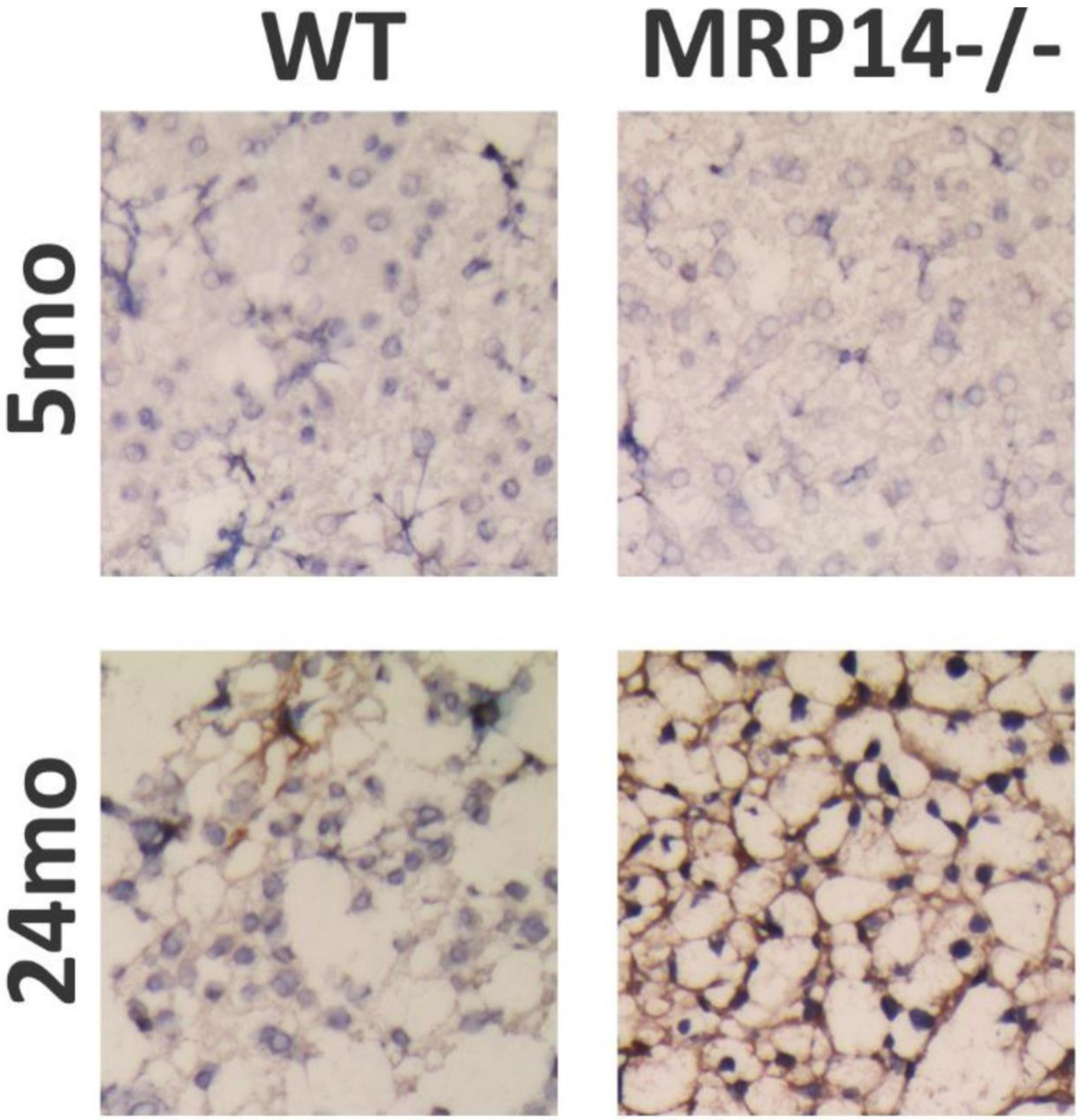

Figure S3. CD45R (B220) antigen staining intensity is strengthened in hepatic tissue from old KO compared to WT mice. Hepatic tissue from young and old WT/KO mice was stained for presence of B lymphocytes using CD45R (B220) antibody.

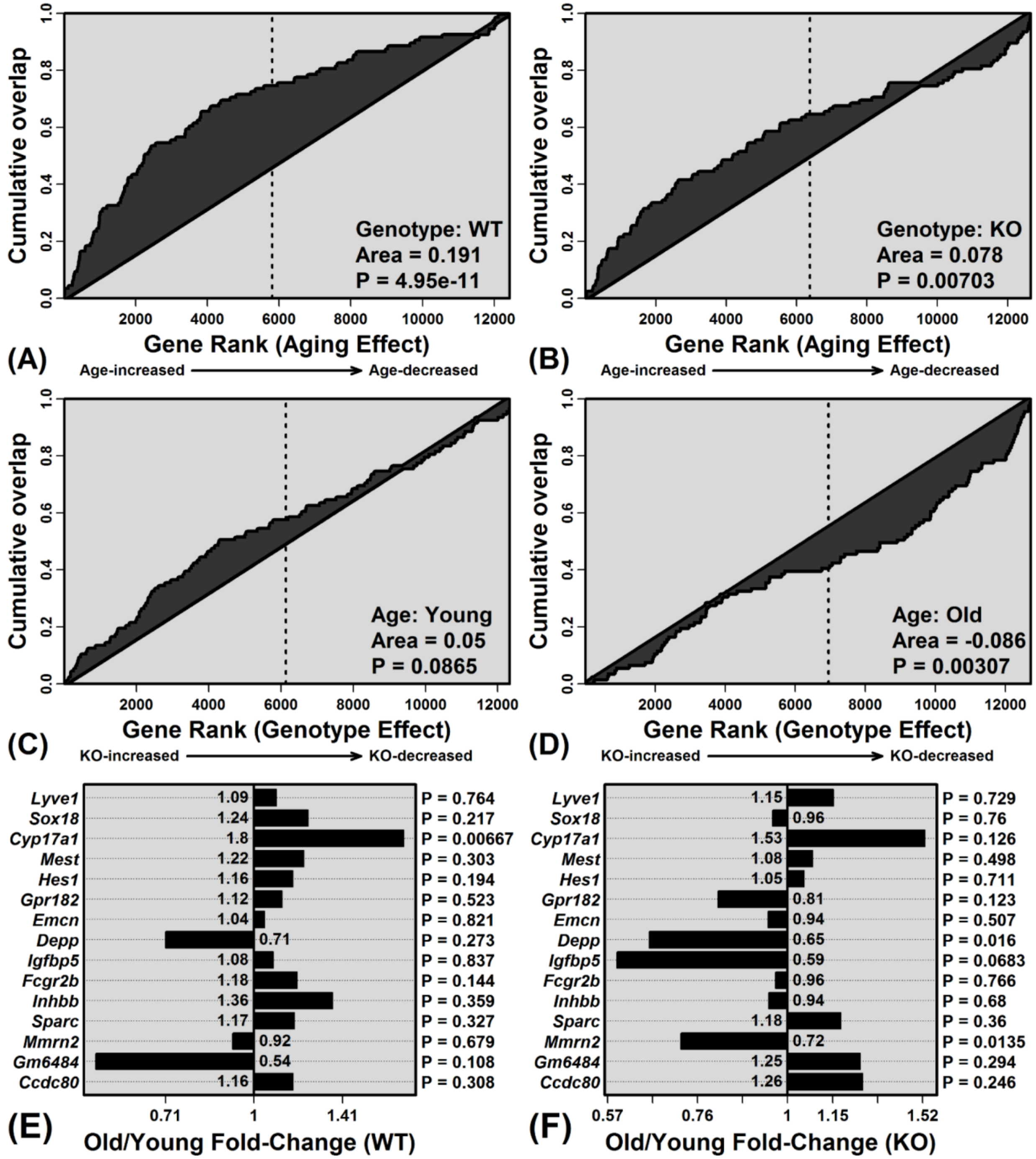

Figure S4. Elevated expression of SRT1720-decreased genes with aging is blunted in KO compared to WT mice. Enrichment of SRT1720-decreased genes (n = 100) among genes with age-increased expression in (A) WT and (B) KO mice. Parts (A) and (B) show cumulative overlap of the SRT1720-decreased genes relative to a list of genes ranked according to how strongly their expression is increased by aging (age-increased: left of vertical line; age-decreased: right of vertical line).

Significant overlap of SRT1720-decreaseed genes and age-increased genes is indicated by a positive area between the cumulative overlap curve and diagonal. Similarly, (C) and (D) show cumulative overlap between SRT1720-decreased genes and ranked according to how strongly their expression is elevated in KO compared to WT mice (C: young mice; D: old mice; KO-increased: left of vertical line; KO-decreased: right of vertical line). Parts (E) and (F) list SRT1720-decreased genes and their FC estimates (old/young) in (E) WT and (F) KO mice. The 15 genes most strongly decreased by SRT1720 in mouse liver are listed (i.e., lowest FC, SRT1720-fed/control).
